# Molecular Dynamics Simulation for the Demulsification of O/W Emulsion under Pulsed Electric Field

**DOI:** 10.3390/molecules27082559

**Published:** 2022-04-15

**Authors:** Shasha Liu, Shiling Yuan, Heng Zhang

**Affiliations:** 1 School of Chemistry and Chemical Engineering, Shandong University, Jinan 250100, China; liushasha325@163.com (S.L.); shilingyuan@sdu.edu.cn (S.Y.); 2 School of Chemistry and Chemical Engineering, Qilu Normal University, Jinan 250100, China

**Keywords:** bidirectional pulsed electric field, O/W emulsion, demulsification, molecular dynamics simulation

## Abstract

A bidirectional pulsed electric field (BPEF) method is considered a simple and novel technique to demulsify O/W emulsions. In this paper, molecular dynamics simulation was used to investigate the transformation and aggregation behavior of oil droplets in O/W emulsion under BPEF. Then, the effect of surfactant (sodium dodecyl sulfate, SDS) on the demulsification of O/W emulsion was investigated. The simulation results showed that the oil droplets transformed and moved along the direction of the electric field. SDS molecules can shorten the aggregation time of oil droplets in O/W emulsion. The electrostatic potential distribution on the surface of the oil droplet, the elongation length of the oil droplets, and the mean square displacement (MSD) of SDS and asphaltene molecules under an electric field were calculated to explain the aggregation of oil droplets under the simulated pulsed electric field. The simulation also showed that the two oil droplets with opposite charges have no obvious effect on the aggregation of the oil droplets. However, van der Waals interactions between oil droplets was the main factor in the aggregation.

## 1. Introduction

With the increase of oil production activities, oil pollution, particularly oily wastewater, has become an environmental concern nowadays. Enormous quantities of oily wastewater are generated during different industrial processes all around the world, including petroleum refining, industrial discharges, petroleum exploration, food production operations, etc. [1,2,3,4,5]. The oils in wastewater include fats, lubricants, cutting oils, heavy hydrocarbons and light hydrocarbons [6]. These oils can be further divided into free oils and emulsified oils. The free oils in wastewater are easier to separate by physical techniques such as gravity separation and skimming [7,8]. However, the emulsified oil droplets are more difficult to handle due to their high stability in water [9,10]. A widely used separation technique for emulsified oils involves the addition of chemicals, such as ferric or aluminum salts, to induce colloidal destabilization [1,5]. However, the cost is expensive, and the chemicals would dissolve in water or form-settling sludge after the treatment, which is not recommended from the perspective of green chemistry.

An alternative approach is the use of an electric field, especially for the dehydration of crude oil [11,12,13,14,15,16,17]. Electric field demulsification has practical advantages such as a lack of extra chemicals, simple equipment, short process flow, etc. It can achieve physical separation of oil and water mixtures and recover oily substances to a certain extent, without the pollution from added chemicals [18,19]. The demulsification mechanism of W/O emulsion by electric fields has also been widely researched. The demulsification was attributed to the droplets’ polarization and elongation under the electric field, which then induces interactions between dipoles, leading to aggregation [20,21,22]. However, the utilization of an electric field to separate oil and water in O/W emulsion is rarely studied. It is generally believed that electric field demulsification does not work for O/W emulsions, because water is conductive, and the electrical energy could dissipate easily in aqueous solution [5]. Ichikawa et al. [23] investigated the demulsification process of dense O/W emulsion in a low-voltage DC electric field and found that a mass of gas bubbles occurred and surged in the emulsion during the demulsification process. Furthermore, Hosseini et al. [24] applied a non-uniform electric field to demulsify the benzene-in-water emulsion. Bubbles were also generated in the emulsion when the electric field was introduced. The occurrence of these phenomena is attributed to the overly large electric current in the emulsion, leading to water’s electrolysis.

To resolve this problem, Bails et al. [25,26] applied a pulsed electric field (PEF) to W/O emulsion and found that the electric current generated by a pulsed electric field is small at a high voltage. After this, Ren et al. [27] applied bidirectional pulsed electric field (BPEF) to separate O/W emulsion; this was prepared by mixing 0 # diesel oil and SDS solution. They found that BPEF induced the aggregation of oil droplets, and BPEF had a distinct demulsification effect on O/W emulsion with surfactant. The demulsification effect under different BPEF voltages, frequencies and duty cycles were investigated by evaluating oil content and turbidity of the clear liquid after demulsification. Moreover, they put forward a hypothesis that charges on the oil drop surface would redistribute under BPEF to promote the mutual attraction and coalescence of oil drops. However, the mechanism of oil droplet movement and aggregation in O/W emulsion at the molecular level under BPEF has not been well studied; still less studied is the effect of surfactants on demulsification. Molecular dynamics (MD) simulation is considered a useful tool to carry out microscopic analysis of the dynamic behavior of nanodroplets based on the basic laws of classical mechanics [28]. Chen et al. [29] used MD simulation to study the influence of a direct current electric field on the viscosity of waxy crude oil and the microscopic properties of paraffin. They found that the electric field strength affects the distribution of oil molecules. He et al. [30] simulated the aggregation process and behavior of charged droplets under different pulsed electric field waveforms by MD simulation. They discovered that the deformation of droplets is greatly affected by the waveform. Moreover, the additive in the emulsion has an important influence on its emulsifying stability [31,32,33]. For example, an experimental study found that BPEF had a distinct demulsification effect on O/W emulsion with SDS surfactant [27]^.^ However, to the best of our knowledge, there has been no report on the microscopic level of the demulsification of O/W systems with SDS surfactants under the action of BPEF electric field. In addition, the crude oil composition was relatively distinct when the behavior of crude oil in electric field was simulated previously [34]. Therefore, it is necessary to study the movement and coalescence behavior of oil droplets in O/W emulsion under BPEF by MD simulation. We believe that this will provide a theoretical basis for the application of BPEF in O/W emulsion demulsification.

In this paper, we investigated the movement and aggregation behavior of crude oil droplets in O/W emulsion with differing contents of SDS under BPEF. First, the structural changes of oil droplets in each system and their collision time were analyzed to determine the behavioral difference between the oil droplets with and without SDS. Second, the centroid distance between oil droplets, the average elongation length of oil droplets and the MSD of SDS and asphaltene molecules of oil droplets were calculated to explain why SDS can reduce demulsification time. Finally, we investigated the aggregation behavior of oil droplets after the shut-off of BPEF and discussed the aggregation mechanism of oil droplets under BPEF.

## 2. Results and Discussion

### 2.1. Emulsified Crude Oil Droplet

**Figure 1:**
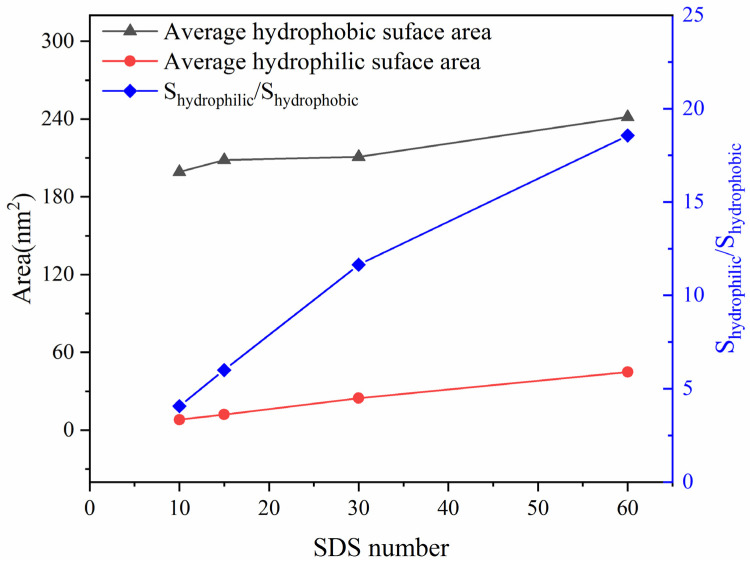
Average hydrophobic and hydrophilic surface areas of emulsified oil droplet.

It was believed that SDS increased the hydrophilicity of oil droplets by increasing the hydrophilic surface area of the droplets [34]. In order to study the surface condition of oil droplets with a different SDS content in each system, the solvent-accessible surface area (SASA) was calculated and shown in Figure 1. With an increasing number of SDS molecules, we noted that both the hydrophilic surface and the hydrophobic surface of crude oil droplets also increased. Meanwhile, the ratio of hydrophilic area to hydrophobic area also increased significantly. Therefore, adding SDS molecules could increase the hydrophilic surface area of oil droplets more significantly. In addition, the greater the number of SDS molecules, the greater the hydrophilic surface area.

### 2.2. Dynamic Behavior of Oil Droplets under BPEF

**Figure 2:**
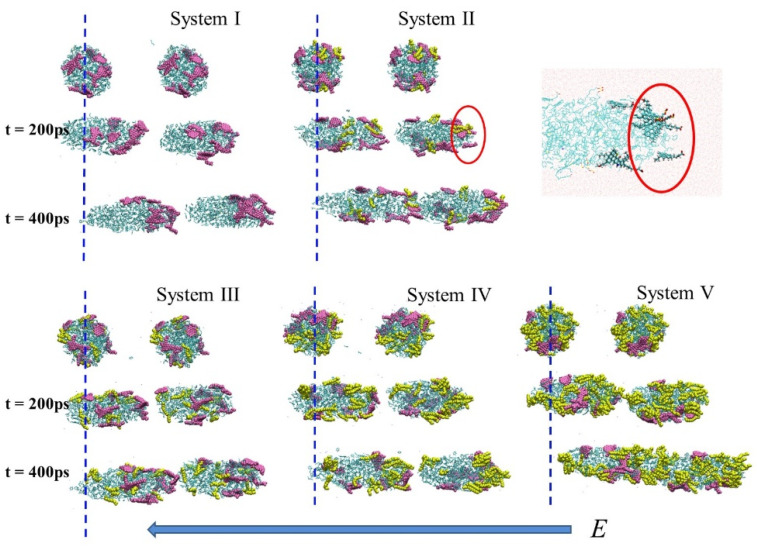
The behavior of oil droplets under BPEF output duration. Asphaltene molecules were colored pink. SDS molecules were colored yellow. Resins and light crude oil molecules were colored green.

In order to study the behavior of oil droplets under electric field, BPEF with *E* = 0.50 V/nm was applied in the z-direction of all systems. Figure 2 displayed the conformational changes of oil droplets with differing SDS content during the electric field output stage. As can be seen from Figure 2, all oil droplets gradually deformed under the electric field, elongated in the z-direction and migrated toward the opposite direction of the electric field. Moreover, SDS and asphaltene molecules concentrated at the end of the oil droplet. Excess SDS molecules were distributed along the entire surface of the deformed oil droplets (System IV, V). To clearly see the distribution of SDS and asphaltene, oil droplets of System II were partly zoomed in. It can be seen that the SDS and asphaltene molecules aggregated at the head of the oil droplet’s moving direction, with the negative sulfonic acid groups of SDS and carboxyl groups of asphaltene molecules facing the opposite direction of the electric field. Therefore, we thought it was the polar SDS and asphaltene molecules that guided the movement of oil droplets under the electric field.

**Figure 3:**
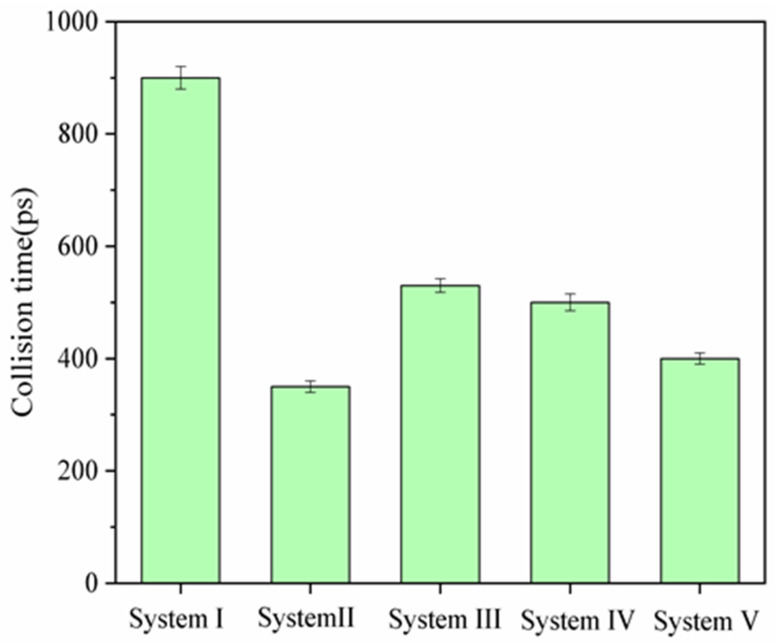
Collision time of oil droplets in five systems under the electric field of 0.5 V/nm.

In Figure 2 we also noted that the states of two oil droplets in five systems were different at 400 ps. In System II and V, the two oil droplets collided at 400 ps. Whereas, for System I, III and IV, this didn’t occur. To investigate the impact of SDS concentration on the coalescence of oil droplets driven by electric field, the collision time was summarized in Figure 3. A collision occurred when the minimum distance between two oil droplets was less than 0.35 nm. It was found that the addition of SDS molecules can reduce the collision time of oil droplets, especially with the 6.2% SDS concentration condition.

### 2.3. Surface Charge Distribution

**Figure 4:**
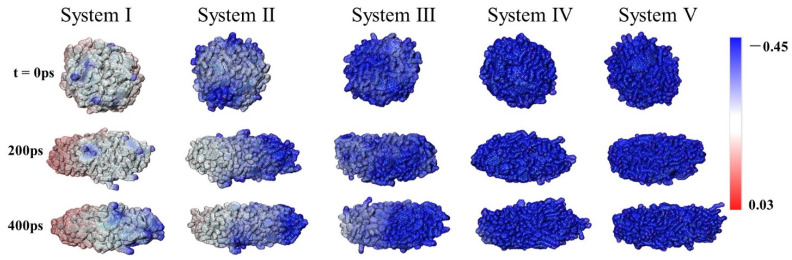
Electrostatic potential surface of the oil droplets during simulation.

**Figure 5:**
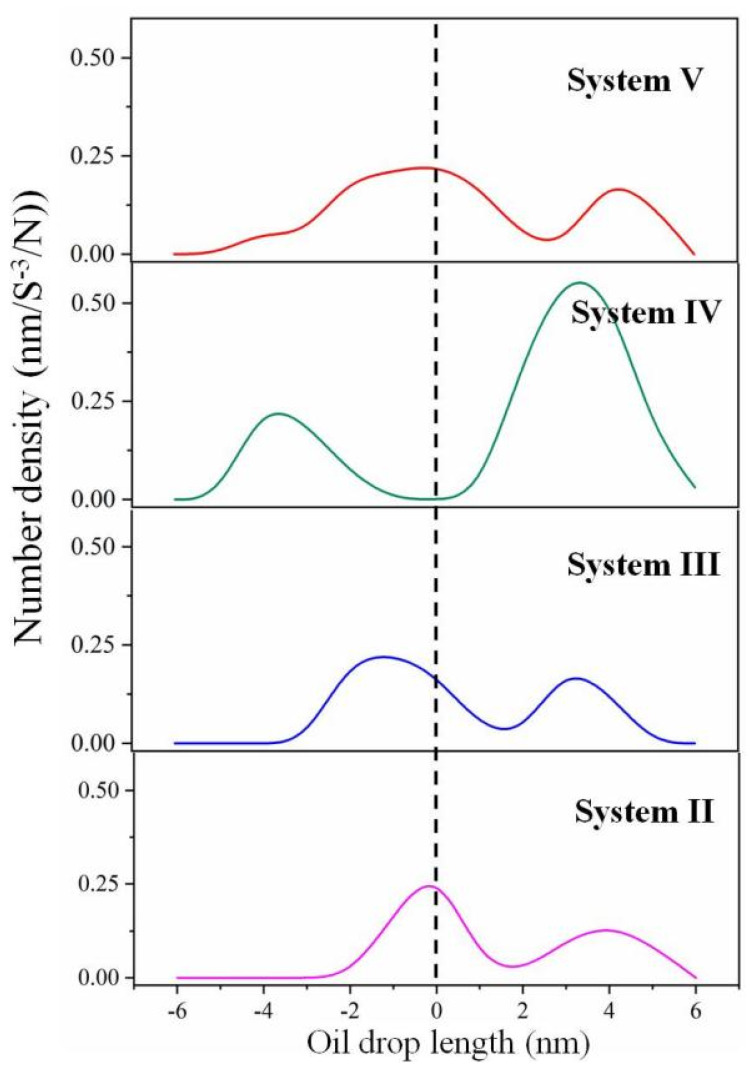
Density profile of SDS in oil droplets under electric field at the same time.

The electrostatic potential surface of the oil droplet can reflect its charge redistribution under electric field. Considering the two oil droplets in each system are the same, only one droplet’s electrostatic potential was calculated. The electrostatic potential diagrams were obtained for different systems at the initial and specific time during the simulation (Figure 4). It can be found that under the influence of the hydrophilic and negatively charged asphaltene molecules on the surface, some areas of the oil droplets appear electronegative (blue area) before electric field is applied. The electronegative area increases with the increase of SDS content. However, the electrostatic potential at the surface of the deformed oil droplet noticeably changed under the electric field, which is manifested as one end of ellipsoidal oil droplet being electronegative toward the opposite direction of the electric field and the other end being electropositive, as in System I and II. These revealed that the redistribution of the charge of oil droplets under electric field resulted in the droplets’ polarization. This phenomenon was consistent with the experimental observation that the charge of the oil droplets under the electric field is positive in the direction of the electric field, and negative in the opposite direction. Meanwhile, we found that for Systems III, IV and V, the oil droplets’ polarization was not obvious. To explain this, the number density of SDS in oil droplets under electric field was analyzed at the same time. In Figure 5, we defined the middle of the oil drop as 0 and the moving direction as the positive direction. It can be seen that with an increase in SDS, it tended to distribute on the surface of the whole deformed oil droplet, which could further explain why the electronegativity area of the deformed oil droplet increased with the increase in SDS.

**Figure 6:**
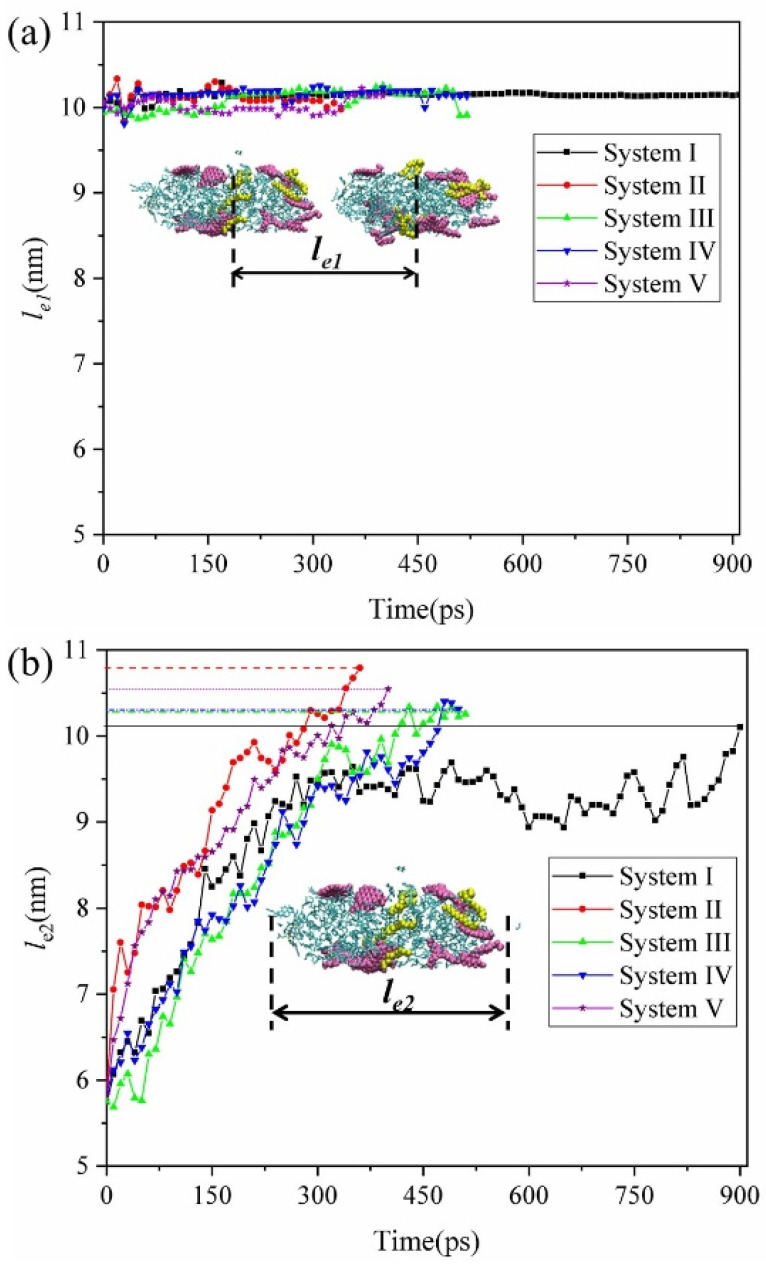
Centroid distance between two oil droplets (**a**) and average elongation length *le* of the two oil droplets along the *z* direction (**b**).

The dynamic behavior of SDS and asphaltene molecules of oil droplets and the electrostatic potential distribution on the surface of oil droplets displayed that the mobile negative charges on oil droplets moved toward the opposite direction of the applied electric field. However, what causes the two oil droplets moving in the same direction to collide, and its relationship with SDS content is unclear. Figure 6 presented the centroid distance between two oil droplets and the average elongation length *le* of the two oil droplets along the *z* direction from the application of the electric field to the collision of oil droplets in each system. We can find that even when the two oil droplets were deformed under electric field, the centroid distance between the two oil droplets remained approximately 10 nm in all systems. This means that due to the oil droplets having the same composition in each system, they moved along the opposite direction of the electric field at almost the same speed, so they kept almost the same initial centroid distance. However, the average elongation length *le* of the two oil droplets in the five systems studied were significantly different. It was found that in all systems, the oil droplets start length was about 6 nm in diameter, and their length increased with time; the average elongation length *le* of the oil droplets exceeded 10 nm near the collision time point. This means that when the length of the oil droplet is stretched enough, the two oil droplets are connected head to end; that is, a collision occurs. Meanwhile, we noted that in Figure 6b, the order of the growth rate of the average elongation length *l_e2_* from largest to smallest is System II, System V, System III≈System IV and System I, which is similar to the trend showing the variation of the collision time of studied systems in this work. Therefore, for the O/W emulsion system with uniform distribution of oil droplets, we thought the demulsification collision time in the electric field is significantly affected by SDS. Adding appropriate SDS surfactant into O/W systems can effectively reduce the power consumption.

**Figure 7:**
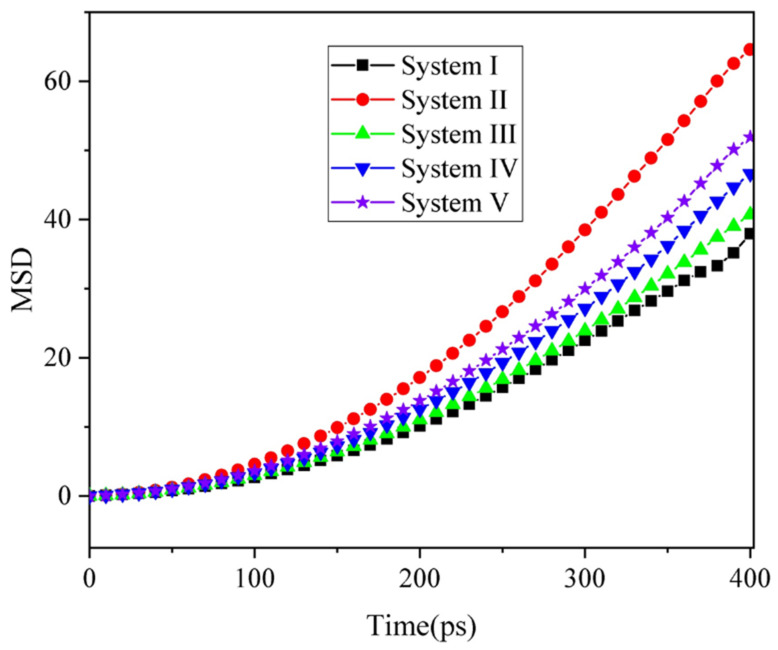
Mean square displacements of SDS and asphaltene molecules in five systems.

As discussed above, SDS and asphaltene molecules guide the entire oil droplet to move in the opposite direction of the electric field. It was predicted that the average elongation length of oil droplets in electric field is related to the diffusivity of the SDS and asphaltene molecules. Thus, we calculated the MSD of SDS and asphaltene molecules for the five systems in Figure 7. It was found that the order of SDS and asphaltene molecules’ diffusion from largest to smallest was System II, System V, System IV, System III and System IV in the five systems studied; this was consistent with the order of the average elongation length of oil droplets under electric field. We thought that in System I the asphaltene molecules interacted more strongly with the surrounding oil molecules due to the influence of its structure, which decreased its mobility under electric field. However, the negative SDS molecules are smaller and demonstrate strong mobility in the electric field, thus increasing their overall mobility. However, this does not mean that the greater the SDS content in the oil droplets, the greater the mobility of negatively charged molecules. Therefore, the SDS content of the oil droplets have great significance on the demulsification effect. Meanwhile, we calculated the root-mean-square fluctuation (RMSF) of oil droplets during the electric field output stage (as shown in Supplementary Materials: Figure S1). By comparing the RMSF of the three systems, we found that the fluctuation of System II and System IV was stronger than that of System I. The addition of SDS could have accelerated the movement of oil droplets, which was similar to the calculation result of MSD.

### 2.4. Aggregation Behavior of Oil Droplets

**Figure 8:**
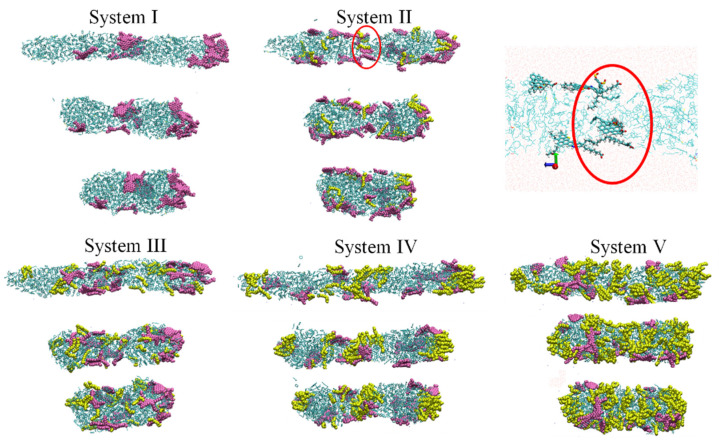
The change in the behaviors of oil droplets during the electric field shut-off period in five systems.

The purpose of electric field demulsification is to aggregate dispersed oil droplets to achieve oil/water separation. Conformations of the oil droplet at the beginning of the collision were selected as the initial structure to simulate the behavior of oil droplets after the shut-off of the electric field (see Figure 8). It can be seen that the oil droplets in contact with each other can continue to aggregate even in the absence of electric field. Taking System II as an example, we found that some asphaltene and SDS molecules, which guided the movement of the oil droplets, formed a contact surface between the two oil droplets and then migrated to the surface of the oil droplets under the influence of hydrophilic groups. At the same time, the hydrophobic components inside the interfacing oil droplets aggregated into a whole. Meanwhile, we calculated the radius of gyration (Rg) during the aggregation of oil droplets in the five systems. (as shown in Figure S2). We found that the radius of gyration of the oil droplets gradually decreased. Therefore, the droplets that collided would gradually aggregate into a whole.

### 2.5. Mechanism of Aggregation of Oil Droplets

**Figure 9:**
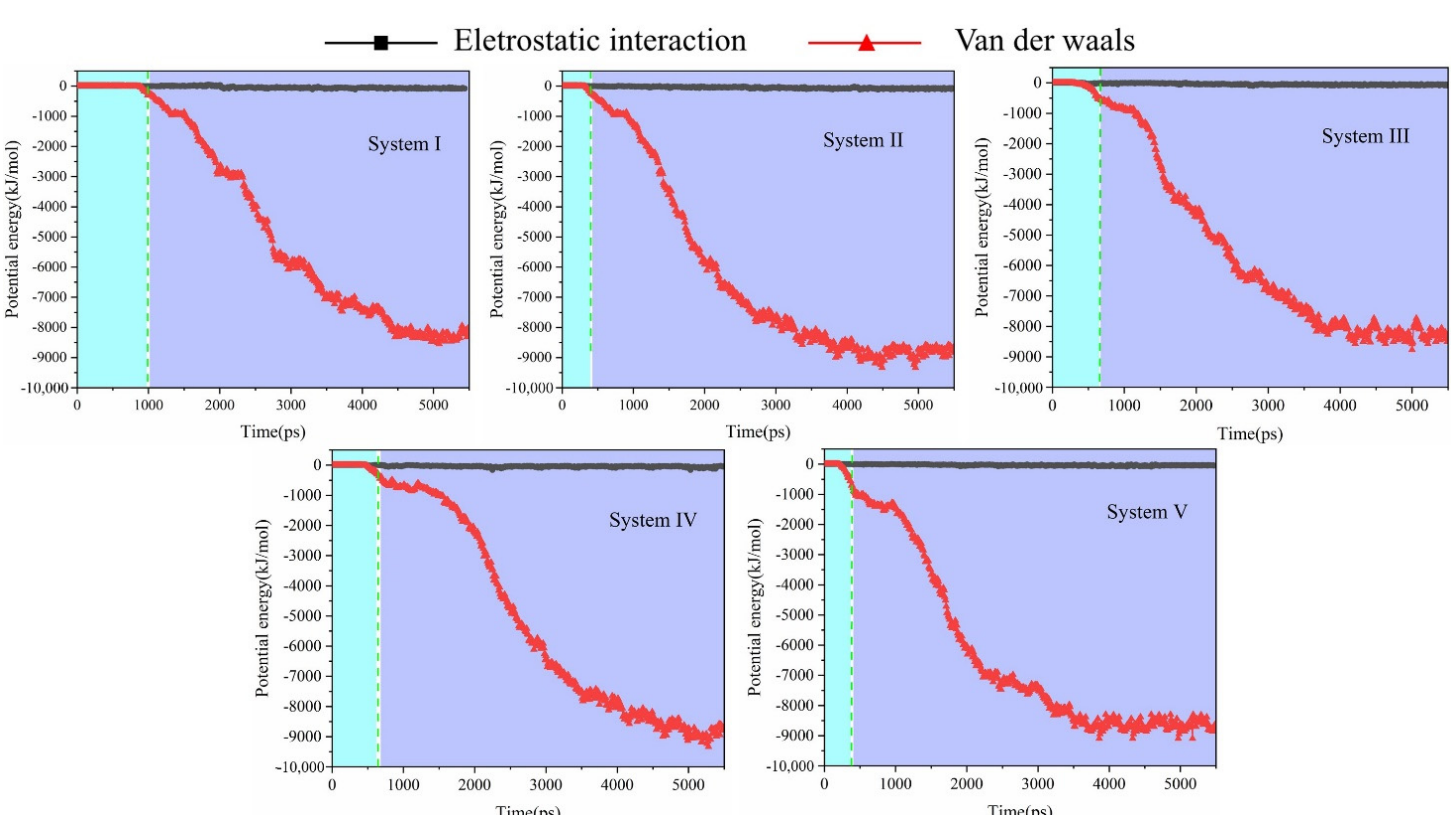
Interaction energy and its decomposed composition between two oil droplets during simulation.

It had been proposed [35] that the surface charges of oil droplets will rearrange under BPEF. The positive and negative charges at the adjacent areas of the two oil droplets are opposite under the action of the electric field. Thus, the adjacent areas of oil droplets always attract each other along the BPEF direction. Here, we verified and explained the accumulation mechanism of oil droplets through theoretical methods. We calculated the interaction energy of the two oil droplets in all systems with *E* = 0.50 V/nm during the whole process from dispersion to aggregation. The calculated results are shown in Figure 9. The potential energy of the interaction between the two oil droplets is divided into two parts. The cyan areas represented the change in the potential energy between the oil droplets from dispersion to collision (i.e., electric field output durations), and the blue areas represented the change in the potential energy with time from collision to aggregation (i.e., electric field shut-off durations). At the same time, we calculated the root-mean-square deviation (RMSD) of crude oil droplets in the five systems (Figure S3). It can be seen from the figure that the aggregated oil droplets were basically stable after 4.0 ns. We found that the potential energy of the electrostatic interaction between the oil droplets was almost 0 kJ/mol during the entire electric field application process. The potential energy of the van der Waals interactions between the two oil droplets from dispersion to collision was also almost 0 kJ/mol in the output electric field stage. However, the potential energy of the van der Waals interactions between the oil droplets noticeably decreased after collision during the aggregation process (electric field shut-off stage). It means that in the electric field demulsification process, the adjacent areas of the two oil droplets with opposite charges have no obvious effect on the attraction and aggregation of the oil droplets. The van der Waals forces between the oil droplets are the main force in the demulsification process.

## 3. Methods and Materials

### 3.1. Simulation Details

All MD simulations were performed in GROMACS 2019.6 software package. The GROMOS 53a6 force field [36] was used. The force field parameters of oil droplet composition were generated by the Automated Topology Builder (ATB) [37,38]. The simple point charge (SPC) model was selected for water molecules. The parameters of sodium ions (Na^+^) that neutralize negative charge have been discussed in the literature [39].

Each system was energy-minimized using the steepest descent method before the simulation. The NVT ensemble at 300 K was performed with velocity rescaling thermostat. The NPT ensemble at 0.1 MPa and 300 K was performed with Berendsen pressure coupling. In the simulation, velocity rescaling thermostat with a time constant of 0.1 ps was selected as the temperature coupling method, and Berendsen pressure coupling with a time constant of 1.0 ps was selected as the pressure coupling method; the isothermal compression factor was set to 4.5 × 10^−5^ bar^−1^. The periodic boundary condition was applied along three dimensions. During the simulation, van der Waals interactions used Lennard−Jones 12-6 potential, and the cutoff was set to 1.4 nm. The Coulombic interaction used particle-mesh Ewald (PME) summation method. The initial velocities were assigned according to Maxwell−Boltzmann distribution. The time step chose 2 fs. The trajectory was saved every 10 ps. VMD 1.9.3 was used for trajectory visualization.

### 3.2. Simulation Systems

#### 3.2.1. Molecular Models of Crude Oil

**Figure 10:**
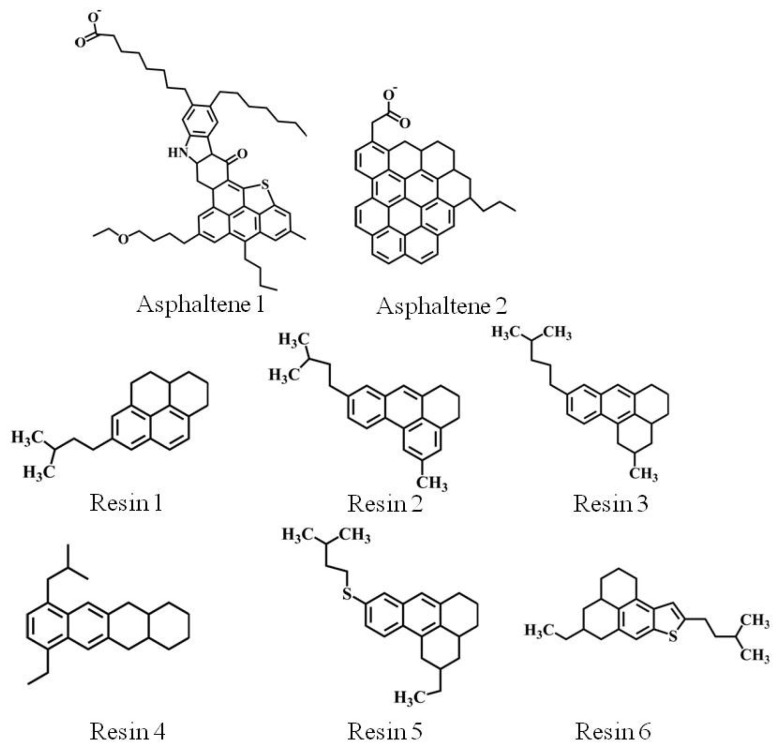
Asphaltene and resin molecules used in simulation.

Owing to the high complexity of crude oil, especially for asphaltene and resins, the asphaltenes demonstrate a key role in the stabilization of water-in-crude oil emulsions and significantly impact the rheological properties of crude oil [40]. Two types of asphaltene (i.e., the number of each type of asphaltene is four) and six types of resins [41] (i.e., the number of each type of resin is five) were selected based on previous studies, as shown in Figure 10. In addition to asphaltenes and resin molecules, four types of alkanes (32 hexane, 29 heptane, 34 octane and 40 nonane molecules), two types of cyclanes (22 cyclohexane and 35 cycloheptane molecules) and two types of aromatics (13 benzene and 35 toluene molecules) were selected as light oil components, referring to Song and Miranda’s work [41,42,43]. Moreover, the concentration of resins and asphaltene in the crude oil was about 38%, which met the content of heavy oil components in crude oil [41].

#### 3.2.2. Emulsified Oil Droplet 

**Figure 11:**
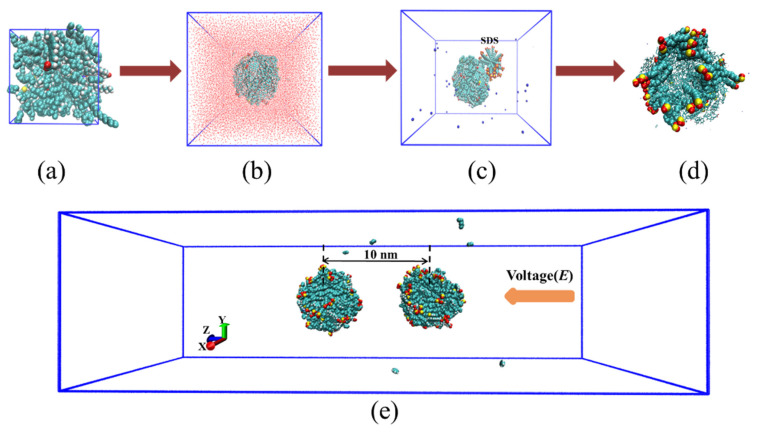
Schematic diagram of the building of emulsified oil droplet. (**a**) Equilibrium configuration of bulk crude oil after NPT simulation, (**b**) emulsified oil droplet in water, (**c**) initial configuration of emulsified oil droplet and SDS micelle, (**d**) Equilibrium configuration of emulsified oil droplet with SDS. For clarity, water molecules in (**c**,**d**) were not shown. (**e**) Lateral view of model simulation for the movement and aggregation behavior of oil droplets in O/W emulsion under BPEF. For clarity, water molecules in the system were not shown.

First, the components of crude oil including alkanes, cyclanes, aromatics, asphaltenes and resins were randomly inserted into a cubic box (x = 10 nm, y = 10 nm, z = 10 nm). To eliminate overlapping, energy minimization was then performed. After that, a 30 ns NPT ensemble simulation was performed to obtain a reasonable density. The equilibrium configuration after NPT run was shown in Figure 11a.

Second, the above crude oil was then solvated in an 8 nm × 8 nm × 8 nm simulation box with 19,230 water molecules. Energy minimization and a 20 ns NVT ensemble MD simulation was carried out to obtain the emulsified oil droplet (Figure 11b). 

Third, emulsified oil droplets with different amounts of SDS adsorbed on their surface was constructed. SDS micelles were constructed using Packmol. The above spherical oil droplets were then placed in the center of a new box (10 nm × 10 nm × 15 nm) and SDS micelles were placed close to oil droplets (Figure 11c). Then, Na^+^ counter ions and solvent were added. After energy minimization and a 20 ns NVT simulation, emulsified oil droplet systems were derived (Figure 11d).

We assumed that the oil droplets distributed in the emulsion had the following conditions: First, the centroids of the two oil droplets were approximately along the *z*-axis direction; Second, the centroids of the two drops were about 10.0 nm apart; Finally, two identical emulsified oil drop models with counter ions were placed in a 10 × 10 × 50 nm^3^ box with a separation distance of about 10 nm, as shown in Figure 11e. Afterward, water molecules were added to solvate the system. The energy minimization and a 10 ns NVT simulation were applied to ensure emulsion system equilibrium. Subsequently, BPEF was imposed on all systems to study coalescence of the two droplets. The composition of each emulsified oil droplet system was shown in Table 1.

## 4. Conclusions

In this paper, molecular dynamics simulations were performed to study the behavior of oil droplets in O/W emulsion. The differences in oil droplets emulsified by different amounts of SDS were compared. Three major conclusions were derived. First, the hydrophilicity of oil droplets increases with increasing SDS content in the oil droplet. When electric field is applied, oil droplets move in the opposite direction of the electric field. The molecules in the oil droplet underwent redistribution. SDS and asphaltene with negatively charged functional groups were transferred to the head of the droplet along the direction of movement. The electrostatic potential surface of the oil droplet proved that the BPEF made the molecules redistribute in the droplet, which resulted in its surface potential redistribution as well. This is consistent with the theoretical hypothesis proposed by this experiment. Meanwhile, the collision time of oil droplets in all simulation systems was different due to the different SDS mass fraction, and the collision time was the shortest for the oil droplets with 6.2% SDS. The average elongation length *le* of the two oil droplets along the *z* direction explained that SDS molecules could change the elongation length of the oil droplets in the electric field. The MSD of SDS and asphaltene molecules under electric field showed that the mobility was the strongest in System II. Therefore, the elongation length of the oil droplets in System II was the largest, and this system was the least time consuming. Second, the oil droplets after collision can self-aggregate after electric field shut-off. SDS and asphaltene molecules on the contact surface between the two oil droplets migrated to the surface of the oil droplets under the influence of hydrophilic groups. Lastly, the adjacent areas of the two oil droplets with opposite charges have no obvious effect on the attraction and aggregation of the oil droplets, and the van der Waals forces between oil droplets are the main force in the demulsification process.

## Figures and Tables

**Table 1:** Details of the emulsified oil droplet systems.

System	Number of Molecules				Mass Fraction (SDS of Oil Droplet)
	Crude Oil Droplet	SDS	Na^+^	Water	
I	1	0	8	47,086	0.0%
II	1	10	18	47,018	6.2%
III	1	15	23	46,985	9.1%
IV	1	30	38	46,883	16.6%
V	1	60	68	46,680	28.5%

## Data Availability

Not applicable.

## Supplementary Materials

The following supporting information can be downloaded at: https://www.mdpi.com/article/10.3390/molecules27082559/s1, Figure S1: Root-mean-square fluctuation (RMSF) of oil droplets in system I, system II and system IV, Figure S2: Radius of gyration (Rg) of oil droplets in five systems, Figure S3: Root-mean-square deviation (RMSD) of oil droplets in five systems.
