# Supplementary material for: Molecular Dynamics Simulation for the Demulsification of O/W Emulsion under Pulsed Electric Field
Source: Molecules. 2022 Apr 15;27(8):2559. doi: 10.3390/molecules27082559 (PMC9029195; doi:10.3390/molecules27082559)
Supplement: Supplementary file 1 [file molecules-27-02559-s001.zip › molecules-1630905-supplementary.pdf]

Supporting Information

Molecular Dynamics Simulation for the Demulsification of O/W Emulsion under Pulsed Electric Field

Shasha Liu [1,2], Shiling Yuan [1] and Heng Zhang [1,*]

[1] School of Chemistry and Chemical Engineering, Shandong University, Jinan 250100, China;
liushasha325@163.com (S.L.); shilingyuan@sdu.edu.cn (S.Y.)
[2] School of Chemistry and Chemical Engineering, Qilu Normal University, Jinan 250100, China
* Correspondence: zhangheng@sdu.edu.cn.

[*a]Corresponding Author, E-mail: zhangheng@sdu.edu.cn.

Tel: +86-531-88366428.

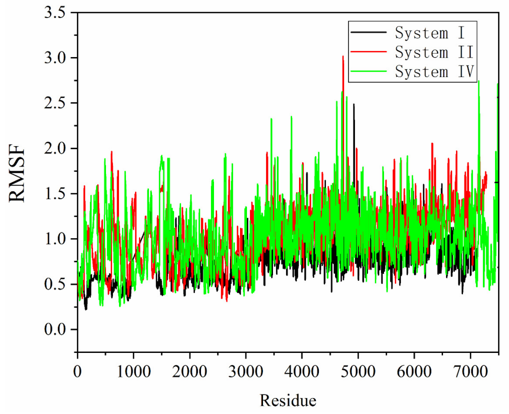

Figure S1. Root-mean-square fluctuation (RMSF) of oil droplets in system I, system II and system IV.

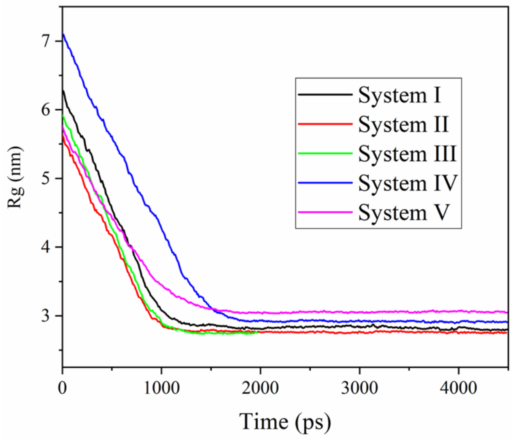

Figure S2. Radius of gyration (Rg) of oil droplets in five systems.

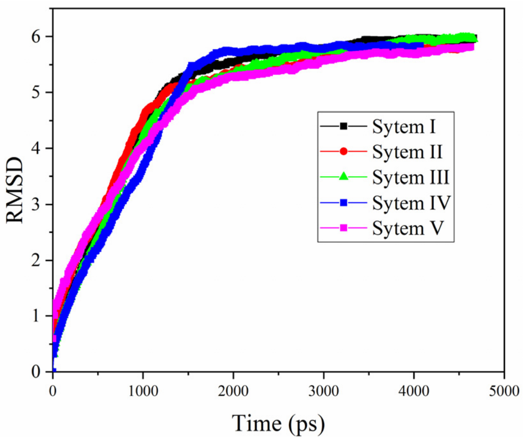

Figure S3. Root-mean-square deviation (RMSD) of oil droplets in five systems.
